# Influence of Ether and Chloroform on the Mind

**Published:** 1855-10-01

**Authors:** 


### INFLUENCE OF ETHER AND CHLOROFORM ON THE MIND.

Dr. Beale, a dentist in Philadelphia, was a short time ago accused and convicted of a gross outrage upon a young lady, upon whom he was performing dental operations, while under the influence of chloroform. The only evidence against him (says the "New York Daily Times") was that of the lady herself; while, on his side, was the weight due to high character, long enjoyed, and never before impeached, the most solemn and emphatic asseverations of innocence, and the established fact, that persons under the influence of chloroform are always out of their senses, and often subject to the strangest fancies and delusions. The conviction is very strong that Dr. Beale's guilt was not proved, and that he is entirely innocent of the heavy charge brought against him. In view of the circumstances attending this case, a meeting of eminent dentists of New York was held, on the 4th inst., to give their experience of hallucinations which had fallen under their observation, from the use of chloroform. Many very remarkable instances were adduced.

Dr. Allen had observed that the patient would frequently insist that the tooth removed under the influence of ether was not out; and nothing but feeling the cavity, or seeing the tooth, would convince them to the contrary.

Dr. Burras related a case of a lady, who placed her brother's hat on her head, put on his coat, and nursed the sofa pillows on her lap, completely forgetting all she had done when she became rational and conscious. A gentleman, on whom he had operated, became outrageously violent, bellowing furiously, and exclaiming, "I've got you now, Bill Brookes." On the effect of the ether passing away, the patient said that he had imagined that he was at home at Portchester, that "Bill" was robbing his money drawer. A young lady protested that the doctor had slapped her in the face; and though contradicted, persisted in her statement, calling for a mirror to show the red spot caused by the alleged blow.

Dr. Barlow had known ladies (of course unconsciously, yet seemingly not so at the moment) divulge their dearest and most delicate secrets, or relate things they would not wish on any account published. He told an amusing story of an Irishwoman, who maintained that during her forced somnolency she had been in Ireland, and saw there her father, mother, and friends. A woman under such circumstances might be quite honest and faithful in her statements; her impressions have all the force of realities.

Dr. Burdell corroborated the statements of the preceding speaker. He had observed that almost invariably there is a strange misconstruction of passing events. He related a very strong instance in point. A gentleman brought a lady to have a tooth removed; he wished her to use ether. After the operation was over, she said, "George, what did you kiss me for? you took advantage of me."

Dr. Burras referred to a case where a man changed the scene of a dentist's surgery into a bedroom, maintained that there was a woman present, and insisted on her being turned out, exclaiming, "What is that woman doing in my bedroom?"

Dr. Castle, of New York, had not used ether for the last three years; was quite convinced of its injurious effects.

Dr. Crowell stated that a young lady came to have her tooth extracted, accompanied by her mother. No sooner was it removed than she angrily complained that he had very rudely kissed her, and no assurance of her mother could convince her to the contrary. So strongly riveted is the impression that, though nine years ago, on his asking her at Saratoga if she had got rid of that foolish impression, she said, "Oh, that's nonsense; you know you did." In another case, a female insisted that he threw her on the floor, and kneeled on her while he removed a tooth. The doctor mentioned other similar illusions.

Mr. Dillingham had always had the presence of the family physician, or a note from him. He did this upon principle. There were always hallucinations, some remembering partially what was passing.

Mr. Francis said, one lady, while under the influence of chloroform, used language of the most ridiculous, profane, and even obscene description. When recovered, she seemed perfectly unconscious of having uttered any improper expression, and apparently not accustomed to its use.

Dr. Hazlett stated, that a young married lady, of a religious disposition, as she felt the influence of the ether, caught him round the neck in the most endearing manner.

Mr. Hurd related a case where, in presence of her husband, a lady, under the influence of ether, followed, or rather chased himself, the operator, round the room, affording most unmistakable signs of perverted feeling, so much so that her mortified husband desired him not to give her any more. Dr. Bennett was also present.

Dr. Parmeley said, one lady thought she had died, and been in hell. He added, "I would not believe the testimony of my own wife as to anything she might relate while she was under the influence of ether or chloroform."

Dr. Puntam took out twenty-eight stumps and teeth from a female. She said she had been much abused—that he had taken her to Jersey city and abused her in the ferry-boat; but, after many attempts to console her, she said she would try to believe what the lady told her who had been present during the operation.

Mr. Robbins, of Jersey city, had seen more than five hundred instances of the employment of ether. In one case a lady, while passing under its influence, wept most bitterly, said he was ill-using her most shamefully, and even after the extraction of the teeth she thought, and still thinks, that some one did abuse her.

Dr. Dressler stated that he had seen several hundred cases; in one of these a young lady persisting he had given her a kiss, and but for the presence of her mother he had no means of proving the contrary.

Several of the speakers (adds the "New York Times") uttered their earnest convictions of the innocence of Dr. Beale of Philadelphia; one of them had known him intimately sixteen years, and was satisfied he was incapable of the crime laid to his charge. Many who did not know him uttered the same opinion. One, who spoke in language as calm as it was energetic, asked whether the indignant denial of a man whose life had been spotless was not of equal, if not of more, value than testimony liable, as they had all seen, to so many cases of unintentional fallacy?

A further scientific discussion, of the same important subject, took place at a second meeting of New York dentists held on the 8th instant. A large number of dentists attended, and, while a good many coincided in their views and experience with speakers at the previous meeting, some reported that they had not found in their practice any instances of mental hallucination remaining after the effects of the ether had passed away.

Dr. Allen (the Chairman) said he was unable to cite, from his own practice, which had been limited, any case bearing for or against Dr. Beale.

Dr. Kingsley had never seen any hallucinations.

Dr. Lord had witnessed various hallucinations, but never saw evidence of amorous excitement.

Dr. Root never saw anything like indecorousness, save slightly in one case. Had seen cases of hallucination which, however, were dispelled by returning consciousness.

At the close of the meeting a petition for Dr. Beale received many additional signatures.

Dr. Brown, chairman of the first meeting, expresses, in a letter to the "New

York Daily Times," his theory respecting Miss Mudge's delusion, as follows:—"My own opinion is, that in the case under consideration, the young lady entered the abnormal state under the impression of a fear that the operator might take advantage of her unconsciousness; that whilst under the influence of ether, this fear was distorted into an actuality; and that this impression was revived, thus distorted, sooner or later, after her perfect restoration to a normal state of mind."

